# Evaluation of Clinical Symptoms Improvement by Cognitive Behavioral Therapy Using a Smartphone Application in Patients with Temporomandibular Disorder

**DOI:** 10.3390/healthcare11101443

**Published:** 2023-05-16

**Authors:** Na-Kyung Hwangbo, Keon-Cheol Woo, Seong-Taek Kim

**Affiliations:** Department of Orofacial Pain and Oral Medicine, Yonsei University College of Dentistry, Yonsei-ro 50-1, Seodaemun-gu, Seoul 03722, Republic of Korea; 1014hwangbo@gmail.com (N.-K.H.); ppathos@naver.com (K.-C.W.)

**Keywords:** cognitive behavioral therapy, digital therapeutics, smartphone application, temporomandibular disorder

## Abstract

Since the start of the 2019 coronavirus pandemic, interest in digital therapeutics (DTx) has increased. Temporomandibular disorder (TMD) fundamentally requires cognitive behavioral therapy (CBT), including physical self-regulation. An application that records TMD pain and parafunctional activities for CBT has recently been developed. However, evidence of the reduction of clinical symptoms in patients via repetitive software-driven CBT is lacking. The purpose of the present study was to evaluate the impact of applications that support CBT regarding the performance of CBT and the improvement of clinical symptoms in temporomandibular joint patients. From 20 October 2020 to 7 January 2021, we randomly assigned 41 participants diagnosed with TMD to control (conventional treatment) and experimental (conventional treatment + application use) groups. We randomly assigned 41 participants diagnosed with TMD to control (conventional treatment) and experimental (conventional treatment + application use) groups. Improvements regarding the number of tender points, mouth opening, visual analog scale score, pain level upon palpation, joint sound, and stress were compared between the two groups. Compared with the control group, the experimental group showed significant improvements in the number of tender points and degree of mouth opening. They also showed improvements in pain level, joint sound, and locking, although not statistically significantly, as compared with the control group. Thus, further studies with a greater sample size need to be conducted to confirm the findings. Nevertheless, our results showed that repetitive cognitive behavioral therapy using a smartphone application can be used as digital therapeutics for temporomandibular disorder patients.

## 1. Introduction

In recent years, medical and dental experts have endeavored to devise and promote various scientific tools, such as the development and verification of new biomarkers to replace pharmaceuticals and the development of quantifiable disease models [1]. In addition, with the rising popularity of social networks, mobile applications, wearable devices, and cloud-based platforms, they have been paying attention to a new form of treatment called “digital health.” Digital health refers to evidence-based behavioral therapies that are provided online and can be used independently or in conjunction with drugs, devices, or other therapies to prevent, manage, or treat medical conditions or diseases, thereby increasing the access to and effectiveness of healthcare. Digital health optimizes patient management and health outcomes. Digital health is suitable for providing personalized and precise treatment and thus has presented the possibility of “Digital Therapeutics” (DTx) by expanding medical services from a limited space to a widespread digital world [2,3]. DTx is one of the subdivisions of digital health and is defined as “evidence-based behavioral treatments delivered online that can increase accessibility and effectiveness of healthcare” [4]. In the clinical field, DTx has been developed to treat chronic diseases, illnesses that require behavioral control (which often require systematic management), and data collection and analysis [5].

Temporomandibular disorder (TMD) is a disease characterized by dysfunction of the temporomandibular joint (TMJ) and the musculoskeletal system of the head and neck. It has a prevalence rate of 10–15% of the general adult population, with most of the patients being female, aged 20–40 years. The prevalence rate among adolescents is approximately 27% [6,7]. Bruxism, which is one of the causes of TMDs and which reduces quality of life for people in the modern age, also has a prevalence of 22–30% for awake bruxism (AB) and 8–15% for sleep bruxism (SB) [8]. Since TMDs and bruxism are strongly associated with stress, eating, and lifestyle habits, the number of patients has been increasing rapidly as society advances. In particular, according to the American Dental Association (ADA), after the outbreak of the 2019 coronavirus pandemic, the observed TMD symptoms in Feb 2021 had increased by more than 62% as compared to the previous year, due to psychological and environmental factors [9,10].

Typically, most TMD patients visit medical experts when their disease is quite advanced. According to Choi et al., as a result of a follow-up study of patients who discontinued treatment without symptomatic improvement resulting from the initial conservative treatment, 36% of the patients required treatment as the disease became chronic [11]. Consequently, it affects the musculoskeletal system, including the neck and shoulders, and can be aggravated by facial asymmetry and trismus [12]. The treatment of TMD mainly consists of conservative treatment, such as behavior control, physical therapy, medication, and occlusal stabilization splints [13]. Despite the ongoing efforts and time taken to treat via conservative treatments, it is difficult to treat the cause, because patients receive limited education within a short period of time when visiting a dentist and should manage their condition themselves. Consequently, the treatment result varies greatly depending on the patient’s understanding and cooperation. As there are limits to one-off treatments and as cognitive behavior therapy (CBT), stress control, muscle relaxation, and posture correction are effective in treating the fundamental cause of TMD, self-directed treatment by the patient is essential [14].

The clinician’s goal for TMD patients is to restore the function of the patient’s TMJ and return the condition to normal by reducing the patient’s pain and parafunctional activities that burden the TMJ [15]. These procedures require patients to undertake “self-care management” [16,17]. However, currently, effective ways of promoting the importance of self-care management, other than giving short instructions on precautions to patients, are lacking. Accordingly, the need for an active method to help treat TMD through repetitive and voluntary behavior control in all places after the treatment has emerged. For this reason, an attempt has been made to treat TMD using smartphone applications. Recently, a prototype version of such a digital health application equipped with minimum functions has been developed to record and recognize pain and parafunctional activities causing TMD and to actively engage the patients to perform and correct those activities. However, studies on such an approach using DTx are very limited in the field of dentistry.

Therefore, the purpose of this study was to evaluate the impact of early-stage digital health applications, incorporating the recognition of self-behaviors and repetitive CBT, on TMD patients in improving the clinical symptoms. The null hypothesis was that there is no significant difference in the improvement of symptoms, such as the degree of pain, degree of mouth opening, and TMJ sound in TMD patients after the use of application based on CBT.

## 2. Subjects and Methods

### 2.1. Subjects and Methods

#### 2.1.1. Subjects of Research

From 20 October 2020 to 7 January 2021, we evaluated patients who were diagnosed with TMD at Yonsei University Dental Hospital of Oral Medicine. We compared 20 patients who received conservative treatment, such as behavioral therapy, drug treatment, and physical therapy, with a smartphone application and 21 patients who received only conventional treatment. Two participants in the experimental group and one participant in the control group were excluded from this study due to voluntary treatment discontinuation. The number of tender points, degree of mouth opening, subjective pain level, pain level on palpation, joint sound, and stress were compared and analyzed. In this study, the control group and the experimental group were randomly assigned. Randomization is a process that provides each participant with the same chance of being assigned to the intervention or control group.

This study was reviewed by the Clinical Research Ethics Committee at Yonsei University Dental Hospital (IRB No. 2–2020-0055). Interviews were conducted in an independent space to protect the privacy of the subjects. During the in-person interviews, the researchers explained to the patients: the purpose and background of the clinical study, information about smartphone application used in the clinical study, procedures or tests that the subjects will undergo in a clinical study that involves invasive methods to the patient’s body, requirements that the subjects must comply with, other treatment methods or types that the subject may choose, and the potential risks and benefits of these treatments, etc. After explaining the study, we obtained consent from subjects, and the data collected during the study were stored on a computer with limited access.

Inclusion Criteria

(1) Male and female participants between the ages of 12 and 65 years old
(2) Participants diagnosed with the following by a specialist at the Department of Orofacial Pain and Oral Medicine at Yonsei University Dental Hospital: Myalgia, local myalgia, myofascial pain, myofascial pain with referral, Arthralgia
(3) Willing and able to comply with the clinical trial protocol
(4) Participants who understand the contents of the study sufficiently and have voluntarily signed the informed consent form
(5) Participants who own a smartphone capable of running the application and are capable of using the application

Exclusion Criteria

(1) Participants who are not diagnosed with temporomandibular disorders (TMD) according to the DC/TMD criteria
(2) Participants with rare muscle diseases such as severe myasthenia gravis, Eaton-Lambert syndrome, and muscular dystrophy
(3) Participants with severe metabolic disorders such as hypertension and diabetes
(4) Participants diagnosed with neurological disorders (such as Parkinson’s disease or Alzheimer’s disease) or psychiatric disorders
(5) Participants who are currently potentially taking skeletal muscle drugs, alcohol, or drugs from other institutions
(6) Participants who are undergoing orthodontic treatment or are wearing other oral devices
(7) Participants who withdraw their consent during the trial
(8) Other participants deemed inappropriate by the study investigators

#### 2.1.2. Method

In this study, we used a smartphone application that focused on identifying pain and inputting pain levels. The application was designed by a specialist in oral medicine. The experimental group received informed consent in a separate space and received sufficient education on how to install and use the application. Each usage of our smartphone application would take approximately 5–10 min per day, and patients were encouraged to use it every day. The contents of our app were equally prepared based on the Management of Temporomandibular Disorders and Occlusion (2013, J.P. Okeson) and a Yonsei University Oral Medicine questionnaire, and the contents were confirmed to be safe to use. 

### 2.2. Smartphone Application

**Figure 1:**
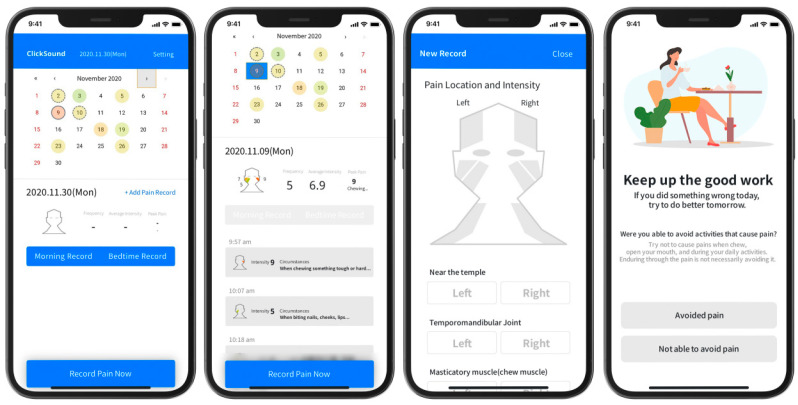
Screenshots showing representative content of the application for self-management of temporomandibular disorder.

This was a minimum viable product (prototype) of Software as a Medical Device (Click Sound Co., Ltd., Seoul, Republic of Korea, August 2020), which is aimed at a TMD DTx. The main functions are (1) Morning Record (morning condition record), (2) Bedtime Record (evening condition record), and (3) Record Pain Now (current condition record, act of recognizing current pain level and inducting CBT). Data from The Record Pain Now function were entered into the calendar and could be checked at any time, and the highest pain score was marked in color on a Visual Analogue Scale (VAS) on the calendar so that the subjective pain score could be seen at a glance. This allowed the patient to grasp the trend of an increase or decrease in pain level (Figure 1).

Types of records

(1) Morning Record
  - Sleep quality
  - Subjective symptoms of bruxism and clenching
  - Pain upon waking up
(2) Record Pain Now
  - Pain site
  - Pain intensity
  - Pain level
(3) Bedtime Record
  - Whether the patient managed to avoid acts that cause pain
  - Whether the patient controlled habitual use of the jaw
  - Whether jaw resting was implemented (‘N’ sound position)
  - Whether jaw health exercise was performed

### 2.3. Acquisition of Clinical Symptoms

#### 2.3.1. Change in the Number of Tender Points

Palpation was performed by a specialist with more than 7 years of experience in orofacial pain treatment. The strength of the force measured was 1 kg for muscles and 0.5 kg for joints, according to the Diagnostic Criteria for Temporomandibular Disorders. The following tender points were evaluated on each side: TMJ, 2 points (lateral, posterior); deep masseter, 1 point; masseter, 3 points (origin, middle, insertion); temporalis, 3 points (anterior, middle, posterior); sternocleidomastoid, 1 point; splenius capitis, 1 point; and trapezius, 2 points (tender point 1, 2). Thus, 13 sites per side (26 sites on both sides) were measured. Discomfort during palpation was recorded, and the conditions before and after treatment were compared.

#### 2.3.2. Degree of Pain in the Chief Complaint Area

**Figure 2:**
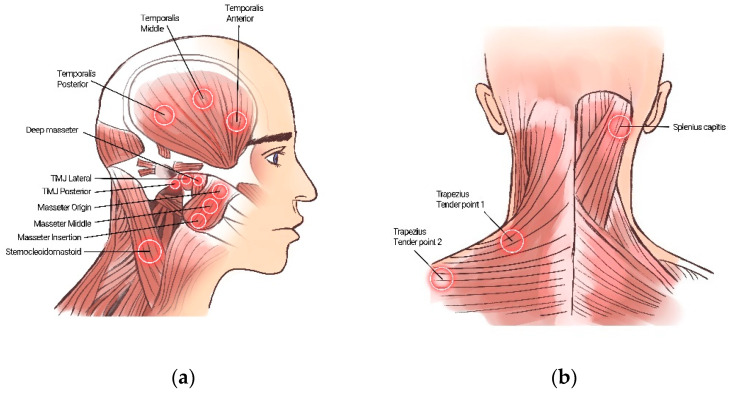
Schematic diagram of the 13 tender points evaluated by palpation (total 26, bilateral). (**a**) Lateral Cervical Region, (**b**) Occipital, Cervical, and Shoulder Region.

Among the 26 tender points, the degree of pain evaluated by palpation of the chief complaint area was subdivided into levels of none, mild, moderate, and severe, which were compared before and after treatment (Figure 2).

#### 2.3.3. Amount of Change in Mouth Opening

**Figure 3:**
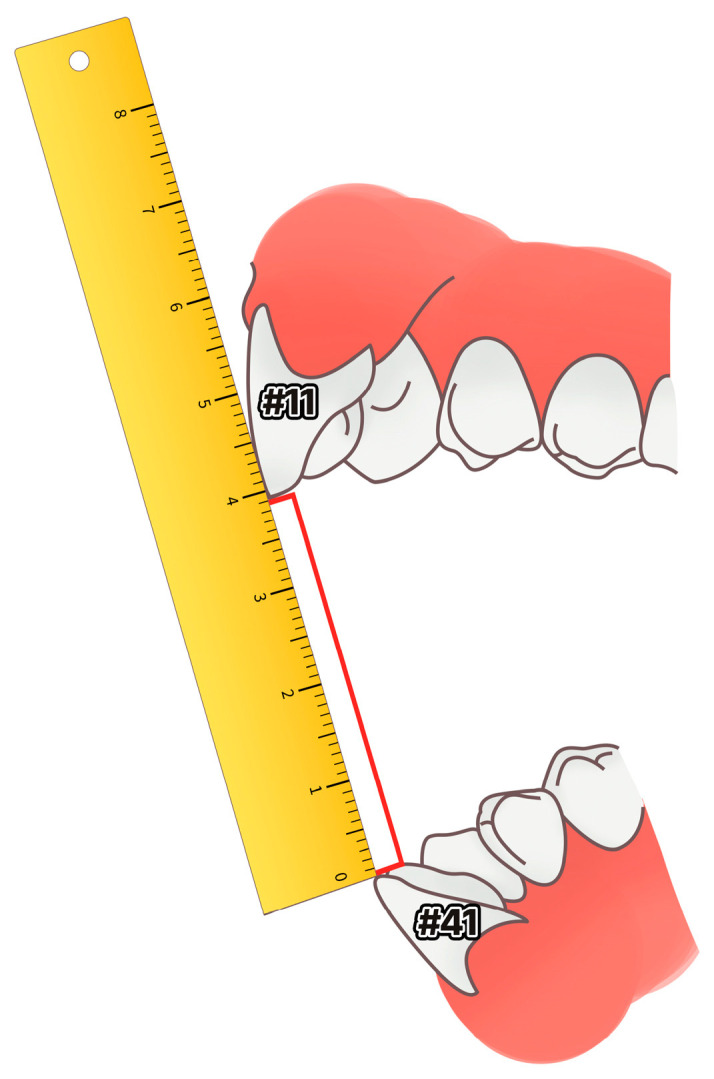
Schematic diagram for measuring maximum mouth opening.

The maximum degree of mouth opening and the other conditions were measured using the distance between the incisors (#11, 41), and were compared before and after treatment (Figure 3).

#### 2.3.4. Measurement of Subjective Pain Level

**Figure 4:**
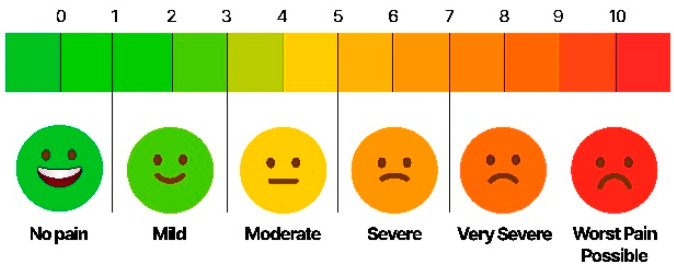
Visual Analogue Scale for Measuring Subjective Pain Intensity.

The overall pain levels according to subjective statements were recorded via a VAS on the day of examination, and the conditions before and after treatment were compared. The criteria for the VAS were 0 point for no pain, 5 points for moderate pain, and 10 points for the worst imaginable pain (Figure 4).

#### 2.3.5. Degree of Change in Joint Sounds and Joint Conditions

Joint sounds from the TMJ were recorded and categorized into none, simple joint sounds (clicking), large joint sounds (popping), and complex joint sounds (crepitus), while the state of locking was recorded separately. A clinician’s auscultation and the patient’s subjective statement of joint sound improvement were recorded before and after treatment.

#### 2.3.6. Degree of Improvement in Stress

We compared the scores calculated on a 5-step Likert scale for 10 questionnaires; detailed information can be found in Supplement 2 (the Korean version of A Global Measure of Perceived Stress, Figure S1).

### 2.4. Statistical Analysis

The data collected in this study were analyzed using IBM SPSS Statistics 25.0 (IBM Co., Armonk, NY, USA), and the statistical significance level was set at *p <* 0.05. The general characteristics of the study subjects were calculated as descriptive statistics and percentages. Differences between the groups were compared using the Mann–Whitney U-test and the chi-square test. Changes in the tender points, degree of mouth opening, VAS scores, stress, and pain levels before and after treatment in each group were analyzed using the Wilcoxon signed-rank sum test. The differences in these variables between the experimental group and the control group were analyzed using the Mann–Whitney U-test. Changes in joint sounds in the experimental group and control group were calculated in percentages and were compared before and after the treatment.

## 3. Results

### 3.1. General Characteristics of Each Group

A total of 41 subjects were randomly assigned to an experimental group (20 persons) and a control group (21 persons). When considering the *p*-values for age, gender, and treatment duration between the two groups, there was no statistical difference. The average duration of treatment for both groups was 5.9 weeks. Their general characteristics are summarized in Table 1.

### 3.2. Changes in Clinical Variables According to Smartphone Application Use

When comparing the changes in clinical variables evaluated before and after treatment, the experimental group showed an average decrease of 5.8 tender points (*p <* 0.001), an average increase of 3.5 mm in the degree of mouth opening (*p =* 0.002), and an average decrease of 3.3 points in the patients’ subjective VAS levels (*p <* 0.001). These differences were statistically significant. In the control group, there was a statistically significant difference regarding the decrease in the number of tender points (mean: 1.8, *p =* 0.002) and the decrease in the patients’ subjective VAS levels (mean: 1.5 points; *p =* 0.012), but there was no significant difference in terms of the increase in the degree of mouth opening or the decrease in stress. In addition, in the experimental group, the number of tender points (*p =* 0.008), mouth-opening degrees (*p =* 0.042), and VAS scores (*p* = 0.012) showed significantly greater effects than in the control group (Table 2).

### 3.3. Change in Pain Level According to Smartphone Application Use

A comparison of the pain level evaluated via palpation of the chief complaint areas before and after treatment showed that both the experimental group and the control group experienced positive treatment effects (Table 3). The experimental group changed as follows: from no group (1 person) to no group (1 person); from moderate group (10 people) to no group (6 people), mild group (2 people), and moderate group (2 people); from severe group (9 people) to no group (1 person), mild group (1 person), and moderate group (7 people). In the control group, the following changes were observed: from no group (3 persons) to no group (3 persons); from mild group (9 people) to no group (3 people), mild group (5 people), and moderate group (1 person); from moderate group (5 people) to mild group (2 people) and moderate group (3 people); from severe group (4 people) to no group (2 people), moderate group (1 person), and severe group (1 person).

### 3.4. Changes of Joint Sound after Using Smartphone Application

When comparing the results of the joint sounds evaluated before and after treatment, one patient’s clicking sound in the control group had changed to no joint sound, but there were no changes in the other joint sounds (Table 4). On the other hand, in the experimental group, one patient’s popping sound changed to a clicking sound, and one patient’s locking state changed to a clicking sound (Table 5). In addition, three patients’ clicking sounds had changed to no joint sounds.

## 4. Discussion

TMD commonly progresses to a chronic stage and can be effectively treated when patient-based repetitive and self-directed behavior-control therapy is combined with conventional treatment. Therefore, although detailed impacts must be evaluated through in-depth study, it is expected that DTx, which can facilitate CBT anywhere conveniently, may be useful for treatment in the appropriate context. In this study, a CBT application was provided as DTx for TMD patients, and its effects were evaluated. Key variables, such as pain level, mouth opening, joint sound, and stress, were assessed. Compared with the control group, the experimental group that used the application in addition to conventional therapy showed significant improvements in those key variables, suggesting that the use of the application could have alleviated the clinical symptoms. Therefore, the null hypothesis was partially accepted.

The application used in this study was developed to promote behavior changes through repetitive CBT, and thereby correct behaviors that were difficult to manage or that were not managed properly through conventional treatments. Forty-one participants were included in the final analysis. The general characteristics of the study participants (age 31.3 years; 24.4% male, 75.6% female) were similar to those of previous studies on TMD prevalence, in which the mean age was 30–40 years, with a ratio of 1:3.3 for men to women [18,19,20,21]. Herein, the experimental group showed overall improvements after application use. The penetration rate of smartphones has already exceeded 100% in developing countries, suggesting that smartphones are a suitable means of DTx, with high accessibility and effectiveness [22]. Additionally, as patients affected with TMD are relatively young, DTx using smartphone applications may have been more effective in this study than it would be for other diseases and age groups [23].

The American Academy of Craniomandibular Disorders states that CBT is an important treatment for TMD [13]. TMD shows a complex interrelationship among the articular disc, masticatory muscles, inflammation, mandibular disorder, and trauma, and CBT influences the entire process. In particular, CBT helps TMD patients to be aware of pain, parafunctional activities, non-functional tooth contact (e.g., clenching), and excessive muscle activity; it is intended to induce a voluntary avoidance and restriction of mouth use to apply therapeutic effects on the fundamental cause. The key elements of CBT are active and voluntary patient participation and confidence that complying with CBT will improve pain [24]. The experimental group underwent routine conventional treatment, including behavior therapy, drug therapy, and physiotherapy, in addition to using the developed application, and showed a mean decrease of 5.8 tender points (*p* < 0.001) while the control group showed a mean decrease of 1.8 tender points (*p* = 0.002). In both groups, the respective treatments showed significant improvements in the number of tender points. Compared with the control group, the experimental group showed a significant decrease in the number of tender points (*p* = 0.008). The mean VAS score (subjective pain level) decreased by 3.3 points in the experimental group (*p* < 0.001) and 1.5 points in the control group (*p* = 0.008). The experimental group showed a significant decrease in the mean VAS score as compared with the control group (*p* = 0.008). The main treatments for myalgia included drug treatments, such as cyclobenzaprine, metaxalone, methocarbamol, and baclofen, as well as non-drug treatment, such as patient education, exercise therapy, CBT, and physical therapy [24,25]. Aggarwal et al. showed that CBT monotherapy, or a combination of CBT and other treatments, effectively improved chronic orofacial pain, such as TMD, in patients [16]. These findings were consistent with our results that showed that repetitive CBT, administered via an application, improved pain in the participants.

The mean degree of mouth opening increased significantly by 3.5 mm in the experimental group (*p* = 0.002). In contrast, the control group showed a mean increase of 1.0 mm in the degree of mouth opening (*p* = 0.072). Compared with the control group, the experimental group showed a significant increase in inter-incisal distance (*p* = 0.042). In general, patients who have inter-incisal distances of less than 35 mm are thought to have mouth-opening limitations, and clinical symptoms may include pain but patients may not feel pain. Mouth opening limitations may present acutely, such as in the form of a myospasm, but they are mostly observed in patients with chronic articular disc displacement (ADD) [6]. In our current study, one patient was diagnosed with disc displacement without reduction and presented a mouth opening limitation. After the treatment, using the developed application, a clicking sound was observed, and mouth opening was improved. This suggests that CBT helps to control bad habits and promote pain relief, muscle relaxation, and disc recapture, which affect the remodeling of the TMJ area [26]. In a study by Nagata, et al., patients with TMD who had mouth opening limitations underwent conventional treatments, consisting of self-exercise, CBT, and management education, and were compared to those who received conventional treatments with a clinician’s manipulations. No significant difference in mouth opening was observed between the two groups. Conventional therapy consisting of CBT was sufficient in improving mouth opening, which was in agreement with our findings [27].

However, in our study, stress did not differ significantly between the experimental and control groups. Stress caused by pain may have decreased by the improvement in temporomandibular pain; however, overall stress, as an external factor, did not change among the participants. As previously described, stress caused by external factors may be a key factor leading to TMD [28]. Thus, updated versions of the application may be helpful if they were to include contents that can reduce mental stress. 

The pain induced by palpation of the tender points labeled as areas of chief complaint was compared before and after treatment. In both the experimental and control groups, positive treatment effects were observed, with no significant differences in the improvements between the two groups. In the experimental group, approximately 45% of the participants showed severe pain before treatment. After conventional treatment, combined with application use, no participant complained of severe pain, and the rate of participants without pain increased significantly from 5% before treatment to 40% after treatment. Similarly, in the control group, the rate of participants without pain increased by 23.8%, and the rate of participants with severe pain decreased by 14.2% after treatment, suggesting that the conventional treatment had positive effects. However, the improvement of the pain after palpation was greater in the experimental group that used the application than in the control group. TMD pain is improved with consistent drug treatments, stretching and posture correction, CBT, physiotherapy, and occlusal stabilization splints [29,30,31]. Our findings showed that voluntary CBT, by using the application, combined with conventional treatment had significant effects in alleviating pain in patients with TMD. 

Overall, joint sound was improved after treatment. In the experimental group, one of the participants with popping sounds reported having clicking sounds after treatment, and three of those with clicking sounds no longer observed joint sounds following treatment. In particular, in one patient with a locking state, joint sound improved to a clicking sound after treatment, which implied encouraging treatment effects. To avoid having to use reduction to treat chronic locking in patients suffering from ADD, education, restricted use and movement range, occlusal splints, and manual manipulation are important. The main goal is to induce the joint to maintain a resting position for as long as possible [24]. CBT using the developed application is thought to maintain the TMJ in a resting position for recovery and healing, improving not only clicking and popping sounds, which are relatively mild, but also locking, which is relatively severe. However, this finding on the improvement of the locking state cannot be generalized as our sample size was limited. Thus, the effects must be validated in further studies with a greater sample size. CBT did not improve the symptoms of those with crepitus. In general, crepitus is associated with osteoarthritic changes in the joint surface [32]. Long-term remodeling effects must be followed-up and evaluated. In the control group, one patient from the clicking group had changed to no joint sounds, but no improvement was shown in other patients.

Liu et al. reviewed five articles on randomized controlled trials (RCTs) and semi-RCTs of 323 studies on the effects of CBT on TMD. According to their report, the reviewed articles resulted in insufficient evidence to present a high grade of recommendation on CBT due to inconsistencies in the methods, such as control groups and follow-up periods. RCTs with more thorough designs were needed [33]. On the other hand, Noma et al. reviewed 18 articles on the effects of CBT on TMD and orofacial pain. CBT monotherapy or combination therapy with other treatments effectively improved the majority of orofacial pain symptoms and recommended a consideration of CBT for the management of orofacial pain by clinicians [34]. Taken together, although long-term additional research and discussion must be presented to better investigate the therapeutic effects of CBT, CBT is thought to have positive effects. Thus, studies must evaluate the effects of long-term CBT. Software-implementing medical devices in the form of applications that support CBT are expected to contribute to DTx. Future studies are expected to expand the scope of CBT via the development of various educational programs that can improve jaw health.

The findings of this study suggested that participating in repetitive CBT by using a smartphone application can improve TMD symptoms. These results may support the development and effectiveness of DTx. Previously, although self-management education and repetitive CBT were noted to be important, there were no options for clinicians to educate and recommended behavioral control after the treatment at the clinics. In this study, partaking in CBT by using a digital application for more than 10 min a day, for 4–6 days a week over 5.9 weeks, helped to control and improve symptoms of TMD. The application is expected to undergo updates focusing on determining professional treatment timing through self-inspection, repetitive learning, and understanding (with remote support), and would present information through data accumulation and analysis. Thus, such an application may be a promising means of DTx for the management and treatment of TMD.

However, some limitations must be considered in the interpretation of this study’s findings. The limited sample size and limited CBT, using only patient records, present challenges in validating the effects of the intervention. Follow-up studies must include a larger sample size and a long-term follow-up period. Additionally, with a larger cohort, the experimental group could be subdivided by diagnosis for a detailed analysis of the effects.

## 5. Conclusions

This study investigated the effects of a smartphone application that helps individuals voluntarily and repeatedly perform CBT for improving clinical symptoms of TMD. Based on the limitations and findings of this study, the following conclusions were drawn. The use of an application supporting CBT showed a positive effect in terms of reducing pain, increasing the degree of mouth opening, and improving joint sounds in patients with TMD, when used in conjunction with conventional treatment methods. These results imply that CBT conducted by using digital applications may be an effective DTx with clinical therapeutic effects for TMD patients. To validate the role of the application as a DTx, the function of the application must be expanded in terms of CBT and it must be tested on larger sample sizes for a longer period of time to demonstrate clinical effectiveness.

## Figures and Tables

**Table 1:** General characteristics according to group.

	Control Group (*n* = 21)	Experimental Group (*n* = 20)	*p*
Age	32.7 ± 13.2	29.9 ± 14.1	0.347 a
Gender			
Male	5 (23.8)	5 (25.0)	0.929 b
Female	16 (76.2)	15 (75.0)	
Treatment period (weeks)	5.9 ± 1.6	5.9 ± 1.9	0.703 a

a Mann-Whitney U-test. Values are presented as mean ± standard deviation. b chi-square test. Values are presented as *n* (%).

**Table 2:** Comparison of changes in clinical variables.

	Control Group			Experimental Group			*p* *^‡^*
	Before	After	*p* *^†^*	Before	After	*p* *^†^*	
Tender points	6.5 ± 4.7	4.7 ± 5.1	0.002	9.0 ± 5.9	3.2 ± 3.3	<0.001	0.008
Mouth opening	45.1 ± 4.4	46.1 ± 4.2	0.072	41.0 ± 7.3	44.5 ± 5.5	0.002	0.042
VAS	4.1 ± 1.1	2.6 ± 2.0	0.012	5.1 ± 1.2	1.8 ± 1.2	<0.001	0.012
Stress	17.1 ± 5.0	17.0 ± 3.8	0.694	15.4 ± 6.6	15.4 ± 7.6	0.924	0.814

*^†^* Wilcoxon signed-rank sum test. *^‡^* Mann-Whitney *U*-test. Values are presented as mean ± standard deviation.

**Table 3:** Comparison of changes in pain level.

	Control Group (*n* = 21)		Experimental Group (*n* = 20)	
	Before	After	Before	After
No	3 (14.3)	8 (38.1)	1 (5.0)	8 (40.0)
Mild	9 (42.9)	7 (33.3)	0 (0.0)	3 (15.0)
Moderate	5 (23.8)	5 (23.8)	10 (50.0)	9 (45.0)
Severe	4 (19.0)	1 (4.8)	9 (45.0)	0 (0.0)

Values are presented as *n* (%).

**Table 4:** Changes in joint sound and state in the control group.

Before	After				
	No	Popping	Crepitus	Clicking	Locking
No (*n* = 7)	7 (100.0)	-	-	-	-
Popping (*n* = 2)	-	2 (100.0)	-		-
Crepitus (*n* = 4)	-	-	4 (100.0)	-	-
Clicking (*n* = 8)	1 (12.5)	-	-	7 (87.5)	-
Locking (*n* = 0)	-	-	-	-	-
Total (*n* = 21)	8 (38.1)	2 (9.5)	4 (19.1)	7 (33.3)	0 (0.0)

Values are presented as *n* (%).

**Table 5:** Changes in joint sound and state in the experimental group.

Before	After				
	No	Popping	Crepitus	Clicking	Locking
No (*n* = 8)	8 (100.0)	-	-	-	-
Popping (*n* = 2)	-	1 (50.0)	-	1 (50.0)	-
Crepitus (*n* = 3)	-	-	3 (100.0)	-	-
Clicking (*n* = 6)	3 (50.0)	-	-	3 (50.0)	-
Locking (*n* = 1)	-	-	-	1 (100.0)	-
Total (*n* = 20)	11 (55.0)	1 (5.0)	3 (15.0)	5 (25.0)	0 (0.0)

Values are presented as *n* (%).

## Data Availability

The data presented in this study are available on request from the corresponding author.

## Supplementary Materials

The following supporting information can be downloaded at: https://www.mdpi.com/article/10.3390/healthcare11101443/s1, Figure S1: A global measure of perceived stress (Korean version).
