# Supplementary material for: Evaluation of Clinical Symptoms Improvement by Cognitive Behavioral Therapy Using a Smartphone Application in Patients with Temporomandibular Disorder
Source: Healthcare (Basel). 2023 May 16;11(10):1443. doi: 10.3390/healthcare11101443 (PMC10218610; doi:10.3390/healthcare11101443)
Supplement: Supplementary file 1 [file healthcare-11-01443-s001.zip › healthcare-2368028-supplementary.pdf]

A global measure of perceived stress

The questions in this scale ask you about your feelings and thoughts during the last month. In each case, you will be asked to indicate how often you felt or thought a certain way by marking with "V."

	0=never, 1=almost never, 2=sometimes, 3= fairly often, 4= very often	◀never				very often▶
1	In the last month, how often have you been upset because of something that happened unexpectedly?	0	1	2	3	4
2	3In the last month, how often have you felt that you were unable to control the important things in your life?	0	1	2	3	4
3	In the last month, how often have you felt nervous and stressed?	0	1	2	3	4
4	In the last month, how often have you dealt successfully with irritating life hassles?	0	1	2	3	4
5	In the last month, how often have you felt that you were effectively coping with important changes that were occurring in your life?	0	1	2	3	4
6	In the last month, how often have you felt confident about your ability to handle your personal problem?	0	1	2	3	4
7	In the last month, how often have you felt that things were going your way?	0	1	2	3	4
8	In the last month, how often you found that you could not cope with all the things that you had to do?	0	1	2	3	4
9	In the last month,, how often have you been able to control irritations in your life?	0	1	2	3	4
10	In the last month, how often have you felt difficulties were piling up so high that you could not overcome them?	0	1	2	3	4

- This questionnaire was modified from A global measure of perceived stress, Cohen, Kamarck, Mermelstein (1983) into Korean standard and is made for Korean university students.
- This content is from "Park Junho, Young Seok Seo (2010), measure of perceived stress for university students (Korean Journal of Psychology 29(3), 611-629". You may visit Korean Psychology website (http://koreanpsychology.co.kr) or KSI, Korean studies information system to see original text.

Figure S1. A global measure of perceived stress (Korean version).
